# The Role of DLNO in the Functional Assessment of Patients with Idiopathic Pulmonary Fibrosis

**DOI:** 10.3390/medicina62010208

**Published:** 2026-01-19

**Authors:** Pasquale Tondo, Josuel Ora, Matteo Pio Natale, Giulia Scioscia, Bartolomeo Zerillo, Matteo Salvatore Di Maggio, Paola Rogliani, Donato Lacedonia

**Affiliations:** 1 Department of Medical and Surgical Sciences, University of Foggia, 71122 Foggia, Italy; pasquale.tondo@unifg.it (P.T.); giulia.scioscia@unifg.it (G.S.); matteosalvodimaggio@libero.it (M.S.D.M.); donato.lacedonia@unifg.it (D.L.); 2 Respiratory and Intensive Care Unit (RICU), Department of Specialistic Medicine, Foggia Polyclinic University-Hospital, 71122 Foggia, Italy; 3 Unit of Respiratory Medicine, Department of Experimental Medicine, University of Rome “Tor Vergata”, 00133 Rome, Italy; josuel.ora@ptvonline.it (J.O.); bartolomeo.zerillo@ptvonline.it (B.Z.); paola.rogliani@ptvonline.it (P.R.); 4 Unit of Respiratory Medicine, Universo Salute Opera Don Uva, 71122 Foggia, Italy

**Keywords:** idiopathic pulmonary fibrosis (IPF), diffusing capacity of the lung for nitric oxide (DLNO), diffusing capacity of the lung for carbon monoxide (DLCO), pulmonary hypertension (PH)

## Abstract

*Background and Objectives:* Idiopathic pulmonary fibrosis (IPF) is a chronic, progressive interstitial lung disease characterized by alveolar-capillary membrane remodeling and impaired gas diffusion. The diffusing capacity of the lung for nitric oxide (DLNO) has been proposed as a physiological parameter reflecting membrane diffusing capacity and pulmonary vascular involvement, potentially providing complementary information to diffusing capacity of the lung for carbon monoxide (DLCO). This study aimed to evaluate the role of DLNO in the functional assessment of patients with IPF and its correlation with clinical and echocardiographic outcomes. *Materials and Methods:* This observational, retrospective study included 35 consecutive IPF patients receiving antifibrotic therapy between February and December 2023. All participants underwent plethysmography, combined single-breath DLNO and DLCO testing, six-minute walk test (6MWT), mMRC dyspnea scale assessment, and echocardiography for the estimation of a higher probability of pulmonary hypertension (PH). *Results:* DLNO was significantly lower in males compared to females (49.3 ± 16.7% vs. 74.6 ± 16.1%, *p* < 0.001), with a reduced DLNO/DLCO ratio in men. DLNO correlated with oxygen therapy requirement (*p* = 0.010) and lower oxygen saturation during the 6MWT (*p* = 0.021). Patients with higher echocardiographic probability of PH showed markedly reduced DLNO values (17.6 ± 7.6%, *p* = 0.016) and higher FVC/DLNO ratios (2.31 ± 0.85 vs. 1.65 ± 0.64, *p* = 0.023), together with lower DLCO levels (*p* = 0.037). *Conclusions:* DLNO may complement DLCO in the evaluation of gas exchange and alveolar-capillary dysfunction in IPF. Although preliminary, these findings support the potential clinical utility of DLNO as an adjunct parameter in the functional characterization of IPF. Further multicenter studies are warranted to confirm these results.

## 1. Introduction

Idiopathic pulmonary fibrosis (IPF) is a chronic, progressive condition characterized by significant morbidity and mortality [1,2]. Patients typically present with persistent dry cough, progressive dyspnea and declining exercise capacity, evidenced by impaired performance in walking tests and progressive hypoxemia in advanced stages. Functionally, IPF manifests as a restrictive spirometric pattern with a marked reduction in alveolar-capillary diffusion.

The alveolar-capillary interface is integral to oxygen transfer, enabling diffusion of oxygen across the alveolar epithelium, interstitial tissue, and vascular endothelium to bind with hemoglobin within erythrocytes. This exchange, governed by Fick’s principles and further elucidated by the Roughton and Forster model, underscores the critical components of alveolar membrane conductance and capillary blood volume (Vc) [3].

The Roughton–Forster equation defines the total resistance to gas uptake (1/DL, where DL is lung diffusion capacity), which is the sum of two primary components: the resistance of the alveolar membrane (1/DM, where DM is membrane diffusion capacity) and the resistance within the red blood cells [1/(θ·Vc), where θ is the rate of gas uptake by hemoglobin and Vc].

Functional impairment in respiratory diseases can be assessed using non-invasive, patient-safe methods such as the diffusing capacity of the lung for carbon monoxide (DLCO) and diffusing capacity of the lung for nitric oxide (DLNO). For carbon monoxide (CO), the majority of the resistance (~70–80%) occurs inside the red blood cells, due to its reliance on hemoglobin binding, while ~20–30% originates from the alveolar membrane. Conversely, for nitric oxide (NO), ~60% of the resistance is attributed to the alveolar membrane and the red blood cell interface, while ~40% occurs within the red blood cells [4].

In IPF, structural alterations at this interface result in the hallmark clinical, functional, and radiological features of the disease [1]. These changes, such as thickening of the interstitial space and fibrosis, primarily increase resistance to gas diffusion across the membrane component (“1/DM”) [5,6]. This imbalance disproportionately impacts the uptake of NO compared to CO, as NO is more sensitive to membrane resistance. The physiological differences between DLCO and DLNO highlight NO’s potential as a parameter sensitive to membrane impairment, particularly in diseases like IPF where membrane integrity is compromised [7]. In IPF, vascular involvement, such as pulmonary hypertension, may also play a significant role in disease progression. Due to the differences in diffusion properties, DLNO and DLCO could better discriminate these vascular changes.

However, measuring DLNO presents technical challenges, including the need for standardized methods, precise equipment calibration, and minimizing variability in test results. Recently, a standardization document published by the European Respiratory Society (ERS) validated this technique for use in respiratory physiology.

To date, no clinical studies have evaluated the role of DLNO in IPF. Previous studies have mainly addressed the role of DLNO in COVID-19 [8]. Given its intrinsic properties, including rapid hemoglobin binding and reduced resistance in the pulmonary circulation compared to DLCO, DLNO has been proposed as a potentially more sensitive indicator of membrane conductance. Whether DLNO provides clinically meaningful additional information in IPF remains unclear. Therefore, we aimed to evaluate the role of DLNO compared with DLCO and explore its correlation with clinical and functional outcomes.

## 2. Materials and Methods

### 2.1. Study Design

This observational, retrospective, single-center study aimed to assess the role of DLNO in IPF patients undergoing antifibrotic therapy using the combined DLNO/DLCO technique. Specifically, the study investigated the correlation between DLNO and clinical, functional, and echocardiographic parameters.

The study was conducted on consecutive IPF patients treated at the Respiratory and Intensive Care Unit, Foggia Polyclinic University-Hospital, Italy between February 2023 and December 2023. All participants underwent comprehensive pulmonary consultations, including respiratory function tests (plethysmography to assess dynamic and static lung volumes, DLCO, and DLNO), a 6 min walk test (6MWT), mMRC dyspnea scale evaluation, and echocardiography to estimate pulmonary arterial pressure systolic (PAPs). Additionally, smoking history and the presence of respiratory failure requiring oxygen therapy were recorded.

The study was conducted according to the International Declaration of Helsinki and approved by our ethics committee. The patients involved in the study signed the informed consent.

### 2.2. Inclusion and Exclusion Criteria

Patients of both sexes aged 18 years or older were eligible for inclusion if they had a diagnosis of IPF, established according to the 2022 ATS/ERS/JRS/ALAT guidelines [2] and were receiving ongoing antifibrotic therapy. Exclusion criteria included the inability to perform respiratory function tests or the 6MWT, hemodynamic instability, uncontrolled arterial hypertension, fever, or recent thoraco-abdominal or ocular surgery within the preceding two months. Any other condition deemed to compromise the safety or feasibility of the tests also resulted in exclusion. All participants were of Caucasian ethnicity, ensuring homogeneity in reference equation applicability.

### 2.3. Respiratory Function Tests

Respiratory function tests, including spirometry, plethysmography, and combined diffusion measurements, were conducted using Vyaire Medical and Jaeger MasterScreen PFT-PRO equipment, SentrySuite software version 3.20.148 (Vyaire Medical, Mettawa, IL, USA). The tests adhered to the ERS recommendations, ensuring standardized measurements [9,10,11,12].

For the diffusion tests, patients inhaled a gas mixture consisting of 14% helium, 0.3% carbon monoxide, 21% oxygen, and nitrogen as the balance gas, with 40 ppb nitric oxide for DLNO measurements. A single-breath maneuver with a 6 s breath-hold was performed with the reaction rate of nitric oxide with hemoglobin (θ) set at 4.5 mL/min/mmHg.

Parameters recorded during the tests included forced vital capacity (FVC), forced expiratory volume in one second (FEV_1_), the FEV_1_/FVC ratio, total lung capacity (TLC), and diffusion indices, including DLNO, DLCO, the DLNO/DLCO ratio, and the FVC/DLNO and FVC/DLCO ratios. Additional measurements included DM, Vc, and alveolar volume (VA). Predicted values and lower limits of normal (LLN) for DLNO, DLCO, and derived ratios were calculated according to the reference equations of Zavorsky et al. [3]. All indices were expressed as absolute values, % predicted, and z-scores, with primary interpretation based on z-scores in accordance with ERS task force recommend.

### 2.4. Statistical Analysis

Statistical analyses were conducted using SPSS software (version 26, IBM, Armonk, NY, USA) and jamovi version 2.7.17. Continuous variables were expressed as mean ± standard deviation, while categorical variables were presented as percentages. The distribution of continuous variables was assessed using the Shapiro–Wilk test to determine normality.

We conducted analyses to assess the differences between the sexes and between patients with a higher versus lower echocardiographic probability of PH (pulmonary hypertension). A systolic pulmonary arterial pressure (PAPS) threshold of 40 mmHg, was used as an echocardiographic screening value to identify patients with a higher probability of pulmonary hypertension, as commonly applied in interstitial lung disease cohorts to selects patients for further diagnostic evaluation, including right heart catheterization. In our cohort, the mean PAPs in the subgroup with higher echocardiographic probability of pulmonary hypertension was close to this threshold, supporting its use for probability stratification [13].

Comparisons between groups were performed using Student’s *t*-test for normally distributed continuous variables and the Mann–Whitney U-test for non-normally distributed variables. Categorical variables were compared using Chi-square tests.

**Figure 1:**
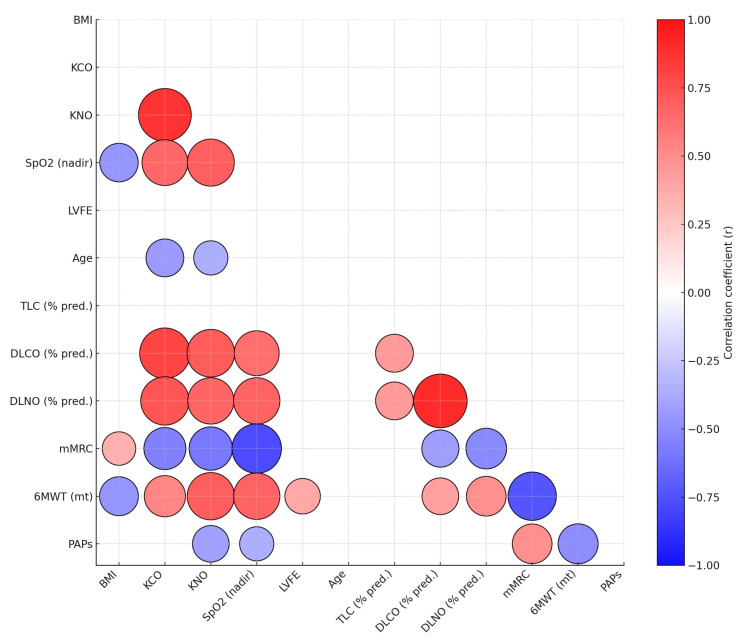
Correlation matrix of main tests parameters.

Correlations between clinical, functional, and echocardiographic parameters were evaluated using Pearson’s correlation test (Figure 1). The parameters analyzed included body mass index (BMI), DLCO, Carbon Monoxide Transfer Coefficient (KCO), DLNO, nitric oxide transfer coefficient (KNO), peripheral oxygen saturation (SpO_2_ nadir), Left Ventricular Ejection Fraction (LVEF), age, TLC % pred., DLCO % pred., mMRC dyspnea score, 6MWT distance (meters), and estimated PAPs. Statistical significance was defined as a *p*-value < 0.05. Given the exploratory nature of the study, *p*-values were interpreted descriptively rather than confirmatorily.

## 3. Results

### 3.1. Demographic and Pulmonary Function Characteristics

The study cohort consisted of 35 participants, with a mean age of 65.8 ± 9.59 years (shown in Table 1 and Table 2). Although males were slightly older than females (67.75 ± 8.58 vs. 61.55 ± 10.71 years), this difference was not statistically significant (*p* = 0.075). The BMI was similar between genders (29.33 ± 4.91 vs. 28.65 ± 4.26, *p* = 0.692), and the prevalence of obesity (BMI ≥ 30 kg/m^2^) was comparable (50% in males vs. 45% in females, *p* = 0.810). A significantly higher proportion of males had a smoking history compared to females (75% vs. 18%, *p* = 0.001).

### 3.2. Pulmonary Function Testing

FEV_1_ (% predicted: 87.58 ± 18.36 vs. 95.09 ± 14.80, *p* = 0.243) and Z-score (−0.80 ± 1.15 vs. −0.37 ± 0.98, *p* = 0.295) were similar between males and females. Similarly, FVC (% predicted: 90.50 ± 17.78 vs. 92.55 ± 14.49, *p* = 0.741) and Z-score (−0.65 ± 1.16 vs. −0.51 ± 0.86, *p* = 0.718) did not differ significantly between the two groups. Vital Capacity (VC) was significantly lower in males when expressed as a percentage of predicted (79.96 ± 17.88 vs. 95.09 ± 13.02, *p* = 0.017), with a significantly lower Z-score (*p* = 0.014). FEV_1_/FVC ratio was significantly lower in males (74.45 ± 9.39 vs. 81.19 ± 7.87, *p* = 0.047), suggesting a more pronounced ventilatory impairment.

TLC, ITGV (Intrathoracic Gas Volume), and RV were similar between males and females when expressed as % predicted (TLC: *p* = 0.222, ITGV: *p* = 0.471, RV: *p* = 0.845) and Z-score (TLC: *p* = 0.190, ITGV: *p* = 0.276, RV: *p* = 0.441), with no significant differences observed between the groups.

### 3.3. Diffusion Capacity Results

The diffusion capacity results are presented in Table 2. DLCO (% predicted) was significantly higher in females compared to males (87.64 ± 21.76 vs. 65.17 ± 21.50, *p* = 0.007), with a corresponding difference in Z-score (*p* = 0.016). KCO values were also higher in females, showing a significant difference for both the absolute value (*p* = 0.004) and Z-score (*p* = 0.040), while the difference in % predicted did not reach statistical significance (*p* = 0.065).

Although VA in terms of % predicted and Z-score was similar between genders (*p* = 0.104 and *p* = 0.288, respectively). Similarly, DLNO (% predicted) was significantly higher in females (74.55 ± 16.06 vs. 49.29 ± 16.72, *p* < 0.001), with 100% of males having DLNO values below the LLN compared to 36% of females (*p* < 0.001). Both LLN and ULN for DLNO were also significantly different between genders (*p* < 0.001), reflecting the application of sex-specific reference equations rather than a true pathological difference. Additionally, KNO was higher in females than in males (*p* = 0.037).

Indices of gas-exchange efficiency revealed significant differences between genders. The FVC/DLCO ratio was higher in males (*p* = 0.007), with 50% of males exceeding a value of 1.5 compared to 0% of females (*p* = 0.003). Similarly, the FVC/DLNO ratio was significantly higher in males (*p* = 0.002), with 71% of males above 1.5 compared to 18% of females (*p* = 0.003). Finally, the DLNO/DLCO ratio was lower in males (0.76 ± 0.11 vs. 0.86 ± 0.13, *p* = 0.016), suggesting a reduced gas exchange efficiency relative to pulmonary capillary blood volume.

### 3.4. Functional Capacity and Oxygenation Results

Functional capacity and oxygenation measures revealed some differences between genders (shown in Table 1). The mMRC score, reflecting dyspnea severity, was higher in males than in females (2.25 ± 0.68 vs. 1.82 ± 0.60), but this difference did not reach statistical significance (*p* = 0.079). Similarly, males demonstrated a shorter 6MWT distance compared to females (342.92 ± 121.39 m vs. 396.36 ± 86.06 m), although this difference was also not significant (*p* = 0.199).

However, the SpO_2_ nadir during the 6MWT was significantly lower in males (91% vs. 95%, *p* = 0.021), indicating poorer oxygenation during exercise. Furthermore, oxygen therapy (OT) was required exclusively by males, with 42% of them on OT, while no females required it (*p* = 0.010).

The LVFE was similar between the two groups (60.00 ± 7.00% vs. 57.00 ± 6.00%, *p* = 0.263). Likewise, PAPs and the higher probability of PH showed no significant differences between males and females (*p* = 0.625 and *p* = 0.667, respectively).

### 3.5. Comparison of Idiopathic Pulmonary Fibrosis Patients with and Without Higher Probability of Pulmonary Hypertension

In this study, IPF patients without and with higher echocardiographic probability of PH were compared across several parameters as shown in Table 3. Demographically, there were no significant differences in age between the two groups, but BMI was significantly higher in the high PH probability group. Spirometry results showed a trend towards lower FEV_1_ and FVC in the high PH probability group, with a significant reduction in the FEV_1_ Z-score (*p* = 0.037). Lung volume measurements, including TLC, RV, and ITGV, showed no significant differences between groups. Exercise tolerance was markedly reduced in PH patients, with a significantly shorter 6MWT distance (245 m vs. 393.7 m, *p* < 0.001) and lower oxygen saturation nadir during the test (87% vs. 93%, *p* = 0.002). The high PH probability group also reported more severe dyspnea, reflected by higher mMRC scores (*p* = 0.001).

Diffusion capacity was notably impaired in patients with higher echocardiographic probability of PH. DLCO was significantly reduced both in absolute terms (*p* = 0.028) and as a percentage of predicted values (*p* = 0.037), with a marked decline in the DLCO Z-score (−3.16 vs. −1.53, *p* = 0.014). DLNO was also significantly reduced (*p* = 0.016), accompanied by a substantial decrease in KCO (71.25% vs. 90.56% predicted, *p* = 0.016). The KCO Z-score further highlighted this impairment (−1.91 vs. −0.54, *p* = 0.01), indicating reduced alveolar-capillary membrane function in this subgroup.

The FVC/DLCO ratio was significantly elevated in the high PH probability group (1.68 ± 0.57 vs. 1.30 ± 0.39, *p* = 0.036), indicating a disproportionate reduction in DLCO relative to lung volume. Although a higher proportion of patients in the high PH probability group had an FVC/DLCO ratio greater than 1.5 (63% vs. 26%), this difference approached but did not reach statistical significance (*p* = 0.058). Similarly, the FVC/DLNO ratio was significantly higher in the high PH probability group (2.31 ± 0.85 vs. 1.65 ± 0.64, *p* = 0.023), reflecting impaired nitric oxide diffusion capacity.

The proportion of patients with an FVC/DLNO ratio above 1.5 was also higher in the high PH probability group (75% vs. 48%), although this difference was not statistically significant (*p* = 0.191). In contrast, the DLNO/DLCO ratio did not differ significantly between groups (0.75 ± 0.16 vs. 0.80 ± 0.11, *p* = 0.333).

## 4. Discussion

Our study suggests that DLNO may represent a potentially valuable tool for identifying, quantifying, and monitoring alveolar-capillary membrane impairment in patients with IPF, emerging as a promising marker compared to DLCO. The reduction in DLNO correlated with worsening respiratory performance, including reduced 6MWT distance, final test saturation, need for home oxygen therapy, and higher mMRC dyspnea scores.

DLCO remains a cornerstone parameter in the functional assessment of IPF and is widely used for screening and prognostic stratification, including pulmonary vascular involvement, as it reflects the combined contribution of alveolar-capillary membrane diffusion and pulmonary capillary blood volume. In this context, DLNO may complement DLCO by providing additional physiological insight into membrane conductance, potentially offering greater sensitivity to early alveolar-capillary alterations associated with pulmonary vascular remodeling. As shown in recent studies, the double diffusion technique combining DLNO and DLCO has been proposed as a valuable physiological approach to improve functional characterization [14,15]. In our cohort, lower DLNO values were descriptively associated with impaired exercise performance, oxygen therapy requirement, and echocardiographic evidence of increased probability of pulmonary hypertension, supporting its potential role as an adjunct functional parameter.

Sex-related differences in DLCO and DLNO were observed and are consistent with sex-specific reference equations and physiological differences in lung volumes. Gas-exchange efficiency ratios (FVC/DLCO and FVC/DLNO) were higher in males, indicating a disproportionate reduction in diffusing capacity relative to lung volumes, which may reflect underlying physiological differences [16]. Lower DLNO values were also associated with greater oxygen desaturation during the 6MWT and with oxygen therapy requirement, supporting the link between membrane impairment and reduced gas-exchange reserve [17].

The lower oxygen saturation at the end of 6MWT observed in men, together with a greater need for home oxygen therapy, suggests lower exercise tolerance in male patients. The association between home oxygen therapy and reduced DLCO and DLNO highlights DLNO’s potential sensitivity as a marker of alveolar-capillary impairment.

These findings may reflect intrinsic physiological differences, such as lung volume distribution, but also external factors, including smoking history, which was more prevalent among men (75% vs. 18%). Although disentangling the effects of smoking-related lung injury from disease specific alveolar-capillary impairment remains challenging, a residual confounding effect of smoking on diffusion measurements cannot be excluded and should be considered when interpreting sex-related differences. The higher BMI observed in patients on oxygen therapy further suggests that obesity may aggravate the reduction in diffusion capacity, underscoring the need for careful clinical management in this subgroup [18].

### 4.1. Pulmonary Hypertension

In our study, we observed that patients with IPF who also higher echocardiographic probability of PH had exhibited a significantly higher FVC/DLCO ratio compared to those with low probability of PH. This finding aligns with previous research indicating that an elevated FVC/DLCO ratio can serve as a marker for vascular involvement in fibrotic lung diseases. For instance, a higher FVC/DLCO ratio was associated with the higher probability of PH in patients with IPF [16]. This suggests that in these patients, there may be a disproportionate reduction in gas transfer capability relative to lung volume, likely due to vascular remodeling and impaired alveolar-capillary membrane function.

Similarly, the FVC/DLNO ratio was also elevated in our high PH probability group, indicating that the impairment in nitric oxide diffusion capacity was more pronounced than the reduction in lung volume.

Interestingly, the DLNO/DLCO ratio did not differ significantly between the two groups. This ratio reflects the relationship between gas transfer mediated by hemoglobin binding (DLCO) and membrane diffusion (DLNO). The lack of difference suggests that both components are proportionally impaired in IPF patients with higher echocardiographic probability of PH, indicating a parallel decline in membrane diffusion capacity and hemoglobin-associated gas transfer. Recent evidence highlights methodological and interpretative challenges in the use of CO- and NO-based diffusing capacities [19,20]. The present findings should be interpreted as hypothesis-generating, aimed at identifying potential physiological signals rather than establishing confirmatory or causal associations.

### 4.2. Limitations

This study is limited by its small, single-center, and retrospective design, as well as by the absence of a healthy control group. Its cross-sectional nature does not allow assessment of longitudinal changes or causal inference. The absence of adjusted analyses and the potential impact of residual confounding further support the exploratory nature of the present investigation. Pulmonary hypertension was estimated by echocardiography, which allows probability stratification but is subject to inherent variability. Future studies integrating DLNO with standardized clinical assessment, advanced echocardiographic right ventricular parameters, circulating biomarkers, and functional classification may help to further clarify its role within multidimensional risk stratification strategies for pulmonary hypertension in IPF.

## 5. Conclusions

In summary, this study supports the hypothesis that DLNO may provide complementary information to DLCO in the functional assessment of patients with IPF, particularly in relation to gas-exchange impairment and pulmonary vascular involvement. These findings support the potential value of DLNO as an adjunct physiological parameter in the characterization of alveolar-capillary dysfunction [21]. However, given the small sample size and monocentric design, the results should be considered exploratory. Overall, this study provides an exploratory physiological framework and a rationale for future, adequately powered and longitudinal investigations. Larger multicenter studies including healthy control groups are needed to confirm the clinical applicability of DLNO in IPF.

## Figures and Tables

**Table 1:** Comparison of clinical parameters in patients with idiopathic pulmonary fibrosis (Total, Female and Male Groups). *p* value refers to the difference between female and male.

Variable	Total (*n* = 35)	Female (*n* = 11)	Male (*n* = 24)	*p*-Value
Age, years	65.8 ± 9.59	61.55 ± 10.71	67.75 ± 8.58	0.075
Height, m	1.65 ± 0.09	1.57 ± 0.08	1.68 ± 0.07	<0.001
Weight, kg	79.06 ± 14.50	70.45 ± 12.82	83.00 ± 13.70	0.015
BMI, kg/m^2^	29.12 ± 4.66	28.65 ± 4.26	29.33 ± 4.91	0.692
Obesity, N (%)	17 (49)	5 (45)	12 (50)	0.810
Smoker, N (%)	20 (57)	2 (18)	18 (75)	0.001
mMRC, unit	2.11 ± 0.68	1.82 ± 0.60	2.25 ± 0.68	0.079
6MWT Distance, meters	359.71 ± 113.05	396.36 ± 86.06	342.92 ± 121.39	0.199
SpO_2_ nadir, %	92 ± 5	95 ± 3	91 ± 5	0.021
OT, N (%)	10 (29)	0 (0)	24 (42)	0.010
LVEF (%)	58.00 ± 6.19	60.00 ± 7.00	57.00 ± 6.00	0.263
PAPs (mmHg)	28.86 ± 11.98	27.36 ± 13.25	29.54 ± 11.59	0.625
PAPs > 40 mmHg (%), N	23.00% (8)	18.00% (2)	25.00% (6)	0.667

Abbreviations: 6MWT: 6-Minute Walk Test; BMI: Body Mass Index; LVEF: Left Ventricular Ejection Fraction; mMRC: Modified Medical Research Council Dyspnea Scale; OT: Oxygen Therapy; PAPs: Pulmonary Arterial Pressure (systolic); SpO_2_ nadir: Peripheral Oxygen Saturation.

**Table 2:** Comparison of functional and gas exchange parameters in patients with idiopathic pulmonary fibrosis (Total, Female and Male Groups). *p* value refers to the difference between female and male.

Variable	Total (*n* = 35)	Female (*n* = 11)	Male (*n* = 24)	*p*-Value
FEV_1_, %pr	89.94 ± 17.46	95.09 ± 14.80	87.58 ± 18.36	0.243
FEV_1,_ Z-score	−0.66 ± 1.11	−0.37 ± 0.98	−0.80 ± 1.15	0.295
FVC, %pr	91.14 ± 16.63	92.55 ± 14.49	90.50 ± 17.78	0.741
FVC Z-score	−0.60 ± 1.07	−0.51 ± 0.86	−0.65 ± 1.16	0.718
FEV_1_/FVC, %	76.57 ± 9.38	81.19 ± 7.87	74.45 ± 9.39	0.047
FEV_1_/FVC Z-score	−0.12 ± 1.28	0.33 ± 1.16	−0.33 ± 1.30	0.159
TLC, %pr	84.24 ± 15.50	89.30 ± 12.42	82.04 ± 16.42	0.222
TLC Z-score	−1.35 ± 1.34	−0.89 ± 0.98	−1.56 ± 1.44	0.190
ITGV, %pr	92.39 ± 25.27	97.30 ± 21.74	90.26 ± 26.83	0.471
ITGV Z-score	−0.49 ± 1.29	−0.12 ± 1.16	−0.66 ± 1.33	0.276
RV, %pr	85.55 ± 35.12	87.40 ± 29.01	84.74 ± 38.05	0.845
RV Z-score	−0.79 ± 1.46	−0.48 ± 1.29	−0.92 ± 1.53	0.441
DLCO, mL/min/mmHg	5.41 ± 1.85	5.67 ± 1.26	5.29 ± 2.07	0.573
DLCO, %pr	72.23 ± 23.75	87.64 ± 21.76	65.17 ± 21.50	0.007
DLCO Z-score	−1.90 ± 1.69	−0.91 ± 1.30	−2.35 ± 1.67	0.016
KCO, mL/min/mmHg	1.22 ± 0.30	1.43 ± 0.15	1.12 ± 0.31	0.004
KCO, %pr	86.14 ± 20.30	95.45 ± 9.74	81.88 ± 22.53	0.065
KCO Z-score	−0.85 ± 1.35	−0.17 ± 0.61	−1.17 ± 1.48	0.040
VA, Liters	4.40 ± 1.02	3.88 ± 0.63	4.63 ± 1.09	0.042
VA, %pr	82.54 ± 15.21	88.73 ± 10.96	79.71 ± 16.22	0.104
VA Z-score	−1.33 ± 1.35	−0.89 ± 1.02	−1.51 ± 1.46	0.288
DLNO, mL/min/mmHg	22.82 ± 7.16	24.08 ± 5.19	22.25 ± 7.94	0.492
DLNO, %pr	57.23 ± 20.16	74.55 ± 16.06	49.29 ± 16.72	<0.001
KNO, mL/min/mmHg	5.17 ± 1.20	5.79 ± 0.92	4.89 ± 1.22	0.037
FVC/DLCO	1.38 ± 0.46	1.08 ± 0.18	1.52 ± 0.48	0.007
FVC/DLCO > 1.5 (%)	34.00% (12)	0.00% (0)	50.00% (12)	0.003
FVC/DLNO	1.80 ± 0.73	1.27 ± 0.22	2.05 ± 0.76	0.002
FVC/DLNO > 1.5 (%)	54.00% (19)	18.00% (11)	71.00% (24)	0.003
DLNO/DLCO	0.79 ± 0.12	0.86 ± 0.13	0.76 ± 0.11	0.016

Abbreviations: DLCO: Diffusing Capacity of the Lung for Carbon Monoxide; DLNO: Diffusing. Capacity of the Lung for Nitric Oxide; FEV_1_: Forced Expiratory Volume in 1 s; FEV_1_/FVC: Ratio of Forced Expiratory Volume in 1 s to Forced Vital Capacity; FVC: Forced Vital Capacity; FVC/DLCO: Ratio of Forced Vital Capacity to Diffusing Capacity of the Lung for Carbon Monoxide; FVC/DLNO: Ratio of Forced Vital Capacity to Diffusing Capacity of the Lung for Nitric Oxide; ITGV: Intrathoracic Gas Volume; KCO: Carbon Monoxide Transfer Coefficient; KNO: Nitric Oxide Transfer Coefficient; RV: Residual Volume; VA: Alveolar Volume.

**Table 3:** Comparison of demographic, clinical, pulmonary function, and exercise test variables between patients with pulmonary artery systolic pressure (PAPs) ≤ 40 mmHg (*n* = 27) and those with higher echocardiographic probability of pulmonary hypertension (PH): PAPs > 40 mmHg (*n* = 8). The groups are classified based on PAPs, measured in mmHg.

Variable	PAPs ≤ 40 mmHg (*n* = 27)	PAPs > 40 mmHg (*n* = 8)	*p*-Value
Age, years	66.48 ± 9.03	63.50 ± 11.69	0.448
BMI, kg/m^2^	28.25 ± 4.92	32.05 ± 1.76	0.041
Obesity (%)	9 (33%)	8 (100%)	<0.001
Smoker (%)	16 (59%)	4 (50%)	0.654
FEV_1_, %pr	93.04 ± 17.38	79.50 ± 14.06	0.053
FEV_1_ Z-score	−0.45 ± 1.08	−1.37 ± 0.93	0.037
FVC, %pr	93.37 ± 17.91	83.63 ± 8.30	0.148
FVC Z-score	−0.46 ± 1.14	−1.08 ± 0.57	0.157
FEV_1_/FVC, %	77.33 ± 8.33	74.02 ± 12.64	0.389
FEV_1_/FVC Z-score	−0.02 ± 1.21	−0.47 ± 1.53	0.386
TLC, %pr	85.16 ± 16.23	81.38 ± 13.51	0.556
TLC Z-score	−1.31 ± 1.42	−1.51 ± 1.09	0.718
ITGV, %pr	92.60 ± 25.77	91.75 ± 25.34	0.936
ITGV Z-score	−0.45 ± 1.34	−0.62 ± 1.21	0.762
RV, %pr	84.32 ± 35.38	89.38 ± 36.38	0.729
RV Z-score	−0.90 ± 1.50	−0.44 ± 1.36	0.444
DLCO, mL/min/mmHg	5.78 ± 1.70	4.17 ± 1.87	0.028
DLCO, %pr	76.74 ± 21.70	57.00 ± 25.43	0.037
DLCO Z-score	−1.53 ± 1.43	−3.16 ± 1.96	0.014
KCO, mL/min/mmHg	1.27 ± 0.27	1.03 ± 0.35	0.04
KCO, %pr	90.56 ± 17.74	71.25 ± 22.45	0.016
KCO Z-score	−0.54 ± 1.14	−1.91 ± 1.53	0.01
VA (L)	4.51 ± 1.04	4.02 ± 0.92	0.235
VA (% predicted)	84.11 ± 15.59	77.25 ± 13.39	0.269
VA Z-score	−1.04 ± 1.35	−2.01 ± 1.19	0.091
DLNO, mL/min/mmHg	24.38 ± 6.38	17.58 ± 7.56	0.016
DLNO, %pr	61.30 ± 17.38	43.50 ± 23.96	0.026
DLNO Z-score	−3.38 ± 1.61	−4.87 ± 2.1	0.001
KNO, mL/min/mmHg	5.51 ± 0.99	4.03 ± 1.18	0.001
FVC/DLCO	1.30 ± 0.39	1.68 ± 0.57	0.036
FVC/DLCO > 1.5, N (%)	7 (26%)	5 (63%)	0.058
FVC/DLNO	1.65 ± 0.64	2.31 ± 0.85	0.023
FVC/DLNO > 1.5, N (%)	13 (48%)	6 (75%)	0.191
DLNO/DLCO	0.80 ± 0.11	0.75 ± 0.16	0.333
mMRC, unit	1.93 ± 0.55	2.75 ± 0.71	0.001
6MWT Distance, meters	393.70 ± 87.71	245.00 ± 118.32	<0.001
SpO_2_ nadir, %	93 ± 5	87 ± 4	0.002
LVEF, %	58 ± 6	57 ± 5	0.606
PAPs, mmHg	23.15 ± 5.83	48.13 ± 4.58	<0.001
OT, N (%)	5 (19%)	5 (63%)	0.015

Abbreviations: 6MWT: 6-Minute Walk Test; BMI: Body Mass Index; DLCO: Diffusing Capacity of the Lung for Carbon Monoxide; DLNO: Diffusing Capacity of the Lung for Nitric Oxide; DLNO/DLCO: Ratio of Diffusing Capacity of the Lung for Carbon Monoxide to Diffusing Capacity of the Lung for Nitric Oxide; FEV_1_: Forced Expiratory Volume in 1 s; FEV_1_/FVC: Ratio of Forced Expiratory Volume in 1 s to Forced Vital Capacity; FVC: Forced Vital Capacity; FVC/DLCO: Ratio of Forced Vital Capacity to Diffusing Capacity of the Lung for Carbon Monoxide; FVC/DLNO: Ratio of Forced Vital Capacity to Diffusing Capacity of the Lung for Nitric Oxide; KCO: Carbon Monoxide Transfer Coefficient; KNO: Nitric Oxide Transfer Coefficient; LVEF: Left Ventricular Ejection Fraction; mMRC: Modified Medical Research Council Dyspnea Scale; OT: Oxygen Therapy; PAPs: Pulmonary Arterial Pressure (systolic); Residual Volume; SpO_2_ nadir: Peripheral Oxygen Saturation; TLC: Total Lung Capacity; VA: Alveolar Volume.

## Data Availability

The data that support the findings of this study are available on request from the corresponding author. The data are not publicly available due to privacy or ethical restrictions.

## Abbreviations

6MWT	6-Minute Walk Test
BMI	Body Mass Index
DLCO	Diffusing Capacity of the Lung for Carbon Monoxide
DLNO	Diffusing Capacity of the Lung for Nitric Oxide
DLNO/DLCO	Ratio of Diffusing Capacity of the Lung for Carbon Monoxide to Diffusing Capacity of the Lung for Nitric Oxide
DM	Membrane Diffusion capacity
FEV_1_	Forced Expiratory Volume in 1 s
FEV_1_/FVC	Ratio of Forced Expiratory Volume in 1 s to Forced Vital Capacity
FVC	Forced Vital Capacity
FVC/DLCO	Ratio of Forced Vital Capacity to Diffusing Capacity of the Lung for Carbon Monoxide
FVC/DLNO	Ratio of Forced Vital Capacity to Diffusing Capacity of the Lung for Nitric Oxide
IPF	Idiopathic pulmonary fibrosis
ITGV	Intrathoracic Gas Volume
KCO	Carbon Monoxide Transfer Coefficient
KNO	Nitric Oxide Transfer Coefficient
LLN DLNO	Lower Limit of Normal for DLNO
LVEF	Left Ventricular Ejection Fraction
mMRC	Modified Medical Research Council Dyspnea Scale
OT	Oxygen Therapy
PAPs	Pulmonary Arterial Pressure (systolic)
PH	Pulmonary Hypertension
RV	Residual Volume
SpO_2_ nadir	Peripheral Oxygen Saturation
TLC	Total Lung Capacity
ULN DLNO	Upper Limit of Normal for DLNO
VA	Alveolar Volume
VC	Vital Capacity
Vc	Capillary Blood Volume

## References

[1] Raghu G., Remy-Jardin M., Myers J.L., Richeldi L., Ryerson C.J., Lederer D.J., Behr J., Cottin V., Danoff S.K., Morell F. (2018). Diagnosis of idiopathic pulmonary fibrosis. An official ATS/ERS/JRS/ALAT clinical practice guideline. Am. J. Respir. Crit. Care Med..

[2] Raghu G., Remy-Jardin M., Richeldi L., Thomson C.C., Inoue Y., Johkoh T., Kreuter M., Lynch D.A., Maher T.M., Martinez F.J. (2022). Idiopathic pulmonary fibrosis (an update) and progressive pulmonary fibrosis in adults: An official ATS/ERS/JRS/ALAT clinical practice guideline. Am. J. Respir. Crit. Care Med..

[3] Zavorsky G.S., Cao J. (2022). Reference equations for pulmonary diffusing capacity using segmented regression show similar predictive accuracy as GAMLSS models. BMJ Open Respir. Res..

[4] Borland C.D.R. (1988). Nitric Oxide and Carbon Monoxide in Cigarette Smoke in the Development of Cardiorespiratory Disease in Smokers. Apollo—University of Cambridge Repository. https://www.repository.cam.ac.uk/handle/1810/238521.

[5] Hamer J. (1964). Cause of low arterial oxygen saturation in pulmonary fibrosis. Thorax.

[6] Kjerulf-Jensen K., Kruhøffer P. (1954). The lung diffusion coefficient for carbon monoxide in patients with lung disorders, as determined by C^14^O. J. Intern. Med..

[7] Barisione G., Brusasco C., Garlaschi A., Baroffio M., Brusasco V. (2016). Lung diffusing capacity for nitric oxide as a marker of fibrotic changes in idiopathic interstitial pneumonias. J. Appl. Physiol..

[8] Imeri G., Conti C., Caroli A., Arrigoni A., Bonaffini P., Sironi S., Novelli L., Raimondi F., Chiodini G., Vargiu S. (2024). Gas exchange abnormalities in Long COVID are driven by the alteration of the vascular component. Multidiscip. Respir. Med..

[9] ATS Committee on Proficiency Standards for Clinical Pulmonary Function Laboratories (2002). ATS statement: Guidelines for the six-minute walk test. Am. J. Respir. Crit. Care Med..

[10] Graham B.L., Steenbruggen I., Miller M.R., Barjaktarevic I.Z., Cooper B.G., Hall G.L., Hallstrand T.S., Kaminsky D.A., McCarthy K., McCormack M.C. (2019). Standardization of spirometry 2019 update. An official American Thoracic Society and European Respiratory Society technical statement. Am. J. Respir. Crit. Care Med..

[11] Stanojevic S., Kaminsky D.A., Miller M.R., Thompson B., Aliverti A., Barjaktarevic I., Cooper B.G., Culver B., Derom E., Hall G.L. (2022). ERS/ATS technical standard on interpretive strategies for routine lung function tests. Eur. Respir. J..

[12] Graham B.L., Brusasco V., Burgos F., Cooper B.G., Jensen R., Kendrick A., MacIntyre N.R., Thompson B.R., Wanger J. (2017). 2017 ERS/ATS standards for single-breath carbon monoxide uptake in the lung. Eur. Respir. J..

[13] Humbert M., Kovacs G., Hoeper M.M., Badagliacca R., Berger R.M.F., Brida M., Carlsen J., Coats A.J., Escribano-Subias P., Ferrari P. (2022). 2022 ESC/ERS Guidelines for the diagnosis and treatment of pulmonary hypertension. Eur. Heart J..

[14] Zavorsky G.S., Agostoni P. (2024). Two is better than one: The double diffusion technique in classifying heart failure. ERJ Open Res..

[15] Zavorsky G.S., Barisione G., Gille T., Dal Negro R.W., Núñez-Fernández M., Seccombe L., Imeri G., Di Marco F., Mortensen J., Salvioni E. (2025). Enhanced detection of patients with previous COVID-19: Superiority of the double diffusion technique. BMJ Open Respir. Res..

[16] Tondo P., Meschi C., Mantero M., Scioscia G., Siciliano M., Bradicich M., Stella G.M. (2025). Italian Respiratory Society (SIP/IRS) TF on Gender Medicine. Sex and gender differences during the lung lifespan: Unveiling a pivotal impact. Eur. Respir. Rev..

[17] Zavorsky G.S., Wilson B., Harris J.K., Kim D.J., Carli F., Mayo N.E. (2010). Pulmonary diffusion and aerobic capacity: Is there a relation? Does obesity matter?. Acta Physiol..

[18] Zavorsky G.S., Hoffman S.L. (2008). Pulmonary gas exchange in the morbidly obese. Obes. Rev..

[19] Zavorsky G.S. (2025). Challenges of DLNO 40 Years After its Invention. Arch. Bronconeumol..

[20] Barisione G., Stanojevic S., Brusasco V. (2026). Zone of z-scores uncertainty in pulmonary function interpretation: A proof-of-concept study from CO and NO lung diffusing capacities. Respir. Med..

[21] Wongkarnjana A., Scallan C., Kolb M.R.J. (2020). Progressive fibrosing interstitial lung disease: Treatable traits and therapeutic strategies. Curr. Opin. Pulm. Med..

